# Local sequence context at *KRAS* codons modulates DNA repair efficiency: insights from molecular dynamics simulations

**DOI:** 10.3389/fmolb.2025.1654434

**Published:** 2025-09-03

**Authors:** James Davies, Georgina E. Menzies

**Affiliations:** Molecular Biosciences Division, School of Biosciences, Cardiff University, Cardiff, United Kingdom

**Keywords:** molecular dynamics, DNA repair, KRAS, BPDE, Rad4

## Abstract

**Introduction:**

Benzo[a]pyrene diol-epoxide (BPDE)-induced DNA adducts contribute to the disproportionate mutagenesis of codon 12 in the *KRAS* gene, driven by preferential DNA damage and impaired repair. Codon susceptibility, however, extends beyond oncogenic hotspots, suggesting that BPDE lesions may serve as biomarkers of individual DNA repair capacity and cancer risk. While the genotoxic effects of tobacco smoke are well characterised, their influence on DNA repair remains underexplored.

**Methods:**

Here, we modelled BPDE-adducted *KRAS* sequences at codons 12 and 14, which have been suggested to exhibit differential repair rates, to assess local helical distortion and its impact on nucleotide excision repair (NER).

**Results:**

We show that BPDE adduction at codon 12 induces distinct DNA distortion compared to codon 14, appearing closer to the canonical DNA structure and therefore potentially evading DNA repair, resulting in altered Rad4 binding and compromised lesion recognition.

**Discussion:**

Our findings link the mutational hotspot at *KRAS* codon 12 to impaired NER and highlight the critical role of local sequence context in repair efficiency. These results provide new insights into the interplay between sequence-dependent DNA structure and repair, with implications for mutation accumulation and cancer development.

## Introduction

Lung cancer is one of the leading causes of cancer related death worldwide with an estimated 2.21 million new cases and 1.80 million deaths per year (Sung et al., 2021). Tobacco smoking remains the most prevalent risk factor in lung cancer, with studies reporting 90% of cases in men, and 70%–80% of cases in women being attributed to smoking (Walser et al., 2008). Although this cause is clear, there remains substantial uncertainty surrounding the molecular basis of mutational bias occurring in smoking individuals. As such, it is crucial to identify the mechanisms that underpin this bias, and isolate the effects that carcinogens, originating in cigarette smoke, may impose on the incidence of these mutations. This information could provide crucial evidence surrounding the aetiology of lung tumorigenesis, an understanding from which novel therapeutic/predictive approaches may be developed.

One family of genes with an interesting mutational signature in lung cancer is the *RAS* gene family. This subset of genes encodes highly conserved monomeric guanosine triphosphate (GTP) binding proteins. The 21-KDa, 188 or 189 amino acid proteins, are crucial for intracellular signal transduction, functioning as molecular switches effecting differentiation and proliferation pathways in a wide array of cell types (Cox and Der, 2010). In mammalian cells, the *RAS* protooncogenes exist in one of three homologs–*HRAS*, *KRAS,* and *NRAS*, each of which demonstrates oncogenic potential in response to somatic mutation. Despite major differences with regards to intron length however, each of the three homologs differ little in terms of exon composition, with 83%–90% amino acid sequence identity maintained throughout the respective homologous (Bryant et al., 2014). *RAS* transcripts are ubiquitously expressed throughout the body, as required by their crucial role in basal cellular function. *KRAS*, however, is by far the dominant homolog which may explain its prevalence in ∼15% of all human cancers, but does not explain its involvement in a high percentage of lung cancer mutations (Forbes et al., 2015).

Three mutation “hotspots” are of particular interest when considering the role of *RAS* family in human cancer. Mutation sites are highly conserved throughout the respective homologs, occurring almost exclusively at codons 12, 13 and 61 in both human and rodent models (Bos, 1989). Structural studies have implicated these mutations in tumour development through compromising the capacity of the final gene product to effectively bind to the GTPase-activating protein, thus confining p21 in a GTP-bound, activated mode. Such events terminally active a kinase signalling cascade, leading to uncontrolled cellular division and therefore tumour development (Liu et al., 2021). Despite the relatively even expression of each *RAS* isoform in lung tissue, the incidence of mutations at *KRAS* codon 12 disproportionally outweigh all other hotspot sites. This has been found true in other cancer types including pancreatic, colon and smoking related lung cancers, for which the incidence of *KRAS* codon 12 mutations is 90%, 50%, and 30% respectively (Le Calvez et al., 2005). Interestingly, however, this specific mutation is most common in current smokers with incidence progressively declining to zero in former and never smokers.

In lung cancer, mutation of *KRAS* codon 12 can be characterised by a G:C > T:A base substitution (Prior et al., 2012). Given that relatively minor exposure to the lung carcinogen benzo(a)pyrene diol epoxide (BPDE) is sufficient to accurately replicate this unique mutation signature, BPDE has long been proposed as the best candidate for the link between smoking and lung cancer. It is well established, however, that the mutagenic activity of BPDE adducts is highly dependent on the complex stereochemistry that arises from their activation (Kim et al., 1995). Feng et al., identified that in normal bronchial epithelial cells, the repair of BPDE adducts was significantly lower at *KRAS* codon 12 when compared to the same position in all other homologs (Feng et al., 2002). Moreover, they also linked *KRAS* with an intrinsic susceptibility to damage by tobacco smoke carcinogens given that hotspots for BPDE binding observed in *KRAS* were absent in both *NRAS* and *HRAS*. Interestingly, this inherent susceptibility for adduct formation was also shown to extend beyond codons of oncogenic potential, with the level of BPDE-DNA binding at *KRAS* codon 14 being similar to that observed at codon 12. Despite this however, the lack of mutation at codon 14 in human cancers is largely attributable to its efficient repair, with *in vitro* studies reporting a 90% clearance after 24 h relative to the 50% observed at codon 12 (Feng et al., 2002). Taken together, these results not only suggest that minor variation in sequence context indeed influences genomic capacity for adduct formation but also identifies DNA repair as a crucial determinant of spectral patterning.

Nucleotide excision repair (NER) is considered the principal pathway used in the removal of bulky lesions such as cyclobutene pyrimidine dimers (CPDs) (Bryant et al., 2014; Forbes et al., 2015; Bos, 1989), photoproducts, or benzo [*a*]pyrene adducts (Schärer, 2013). In humans, a sub-pathway of NER, termed global genome (GG)-NER, relies on dedicated lesion recognition factors such as the XPC-RAD23B-CETN2 complex (XPC) to effectively repair lesions present in the genome (Wood, 1999; Puumalainen et al., 2016). XPC is widely considered to be indispensable in GG-NER given its ability to stimulate repair through responding to the unique biophysical properties that are presented at each lesion site (Mu et al., 2015). The associated thermodynamic instability of a lesion bearing duplex renders the energy barrier needed to induce the formation of an open complex lower than that in undamaged DNA (Chen et al., 2015). This energetic accessibility is sufficient to stall the freely diffusing protein at the lesion site long enough for repair to take place. As such, thermodynamic instability forms the basis of a rapid yet precise kinetic gating mechanism by which lesion recognition may take place without the wasteful interrogation of the predominantly nonspecific undamaged genomic background. Many studies, both experimental and theoretical, have eluded to the influence of local sequence context on the efficacy of lesion recognition by NER (Cai et al., 2010). As such, investigating the direct interactions that occur between lesion recognitions factors and BPDE adducts formed among variable sequence contexts may serve as a direct measure of repair sensitivity.

The influence of XPC in human health and cancer is unequivocal. *In vitro* studies attribute the importance of XPC in DNA repair to its ability to recognise and bind an array of bulky lesions, including polycyclic aromatic hydrocarbons (e.g., benzo [*a*]pyrene) with impressive specificity (Trego and Turchi, 2006; Batty et al., 2000). Notably, however, a high-resolution structure of a mammalian XPC is still lacking. Crystal structures of its yeast ortholog, Rad4–Rad23 (hereafter Rad4) bound to DNA model lesions (a TTT/TTT mismatch bubble and a TTT/TTT enclosing a CPD lesion) have been resolved (Min and Pavletich, 2007). Given their high degree of evolutionary conservation from yeast to humans, such structures have revealed considerable similarities in terms of their overall architecture and biochemical specificity (Min and Pavletich, 2007; Krasikova et al., 2013). As such, it is possible to utilise Rad4 as an alternative model to predict a generalised binding mechanism undertaken by XPC in the repair of BPDE adducts.

The Rad4 structure used in this study contains the transglutaminase-like domain (TGD) and β-Hairpin domains 1-3, including the β-hairpins 1–3. The first β-Hairpin domain (BHD1), along with the TGD domain bind to the undamaged part of the DNA and BHD’s 2 and 3 bind to the four base pair fraction of the DNA where the adduct or damage site is found (Kong et al., 2016). In addition, there are multiple contacts between Rad4 and the rest of the crystallised DNA. The β-hairpin from the BHD3 is inserted into the DNA double helix at the damage site causing the DNA to further break it’s Watson-Crick formation, this and the other contacts cause a kink in the DNA (Min and Pavletich, 2007).

It has been shown that important residues, including those with stabilising roles in the structure or those which bind to DNA are highly conserved. Indicating Rad4/XPC having similar roles in recognising damaged DNA this is further expanded upon in Min et al, notably in their Supplementary Figure S2. (Min and Pavletich, 2007). Specifically, they show that the BHD2 and 3 residues involved in DNA binding are conserved between the yeast Rad4 and the human XPC. Broyde *et al* further showed that although human XPC is predicted to have more extensive contacts with the minor groove around the DNA damaged site that BHD3 is similarly associated with the DNA and key contacts remain the same (Mu et al., 2017). Others have shown a strong link between XPC and the Rad4 proteins, making it an appropriate comparison for our on-going analysis (Velmurugu et al., 2016; Geacin et al., 2017; Nem et al., 2015).

To effectively evaluate the role of sequence context on the poor repair efficiency of BPDE adducts formed at *KRAS* codon 12, a fundamental appreciation of the structure and dynamics of such systems is required on the atomic level. Given this small scale, assessing these dynamics by experimental means is simply unattainable. No study has simultaneously analysed the direct relationship between helical distortion and NER repair, factors that are both renowned to regulate nucleobase mutability. Moreover, the majority of existing literature surrounding BPDE adduct repair utilise well-repaired stereoisomers, including that of the 10*R*-(+)-*cis-anti*-benzo [*a*]pyrene-*N* ^2^-dG adduct (Mu et al., 2017; Mu et al., 2018). Consequently, in an effort to evaluate the role of such adducts on spectral patterning, it is crucial to consider an isomer which demonstrates both elevated mutagenic potency and physiological abundance. Of all possible BPDE configurations, (+)-enantiomers represent the most mutagenic compounds within the context of mammalian cell systems, with the 10*S*-(+)-trans-anti-BPDE-N^2^-dG adduct (hereon referred to as 10S) shown to be the dominant form (>90%) (Lee et al., 2014).

**FIGURE 1:**
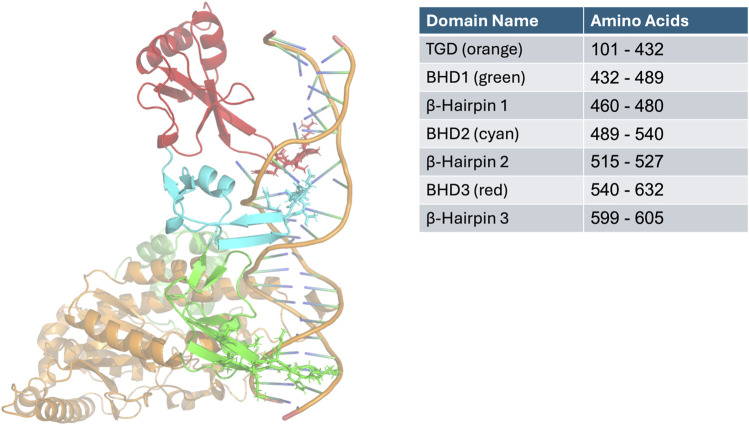
A representation of Rad4 with a KRAS DNA sequence. Domains are visualised in colours corresponding and table with hairpin’s represented with sticks. The right hand table contains domain amino acids number information.

To explore this, we used molecular dynamics (MD) simulations to examine how sequence context influences BPDE-induced helical distortion and NER efficiency. Focusing on the mutagenic (K)-7S,8R,9R,10S + anti-B(a)PDE (10S) adduct, a known G>T mutator and we hypothesise that structural distortions at *KRAS* codon 12 impair NER binding, driving its high mutation frequency in smokers. Our findings, supported by the yeast repair model Rad4, Figure 1, reveal that sequence-dependent helical changes disrupt NER, providing a mechanistic explanation for the mutational bias observed in smoking-related lung cancers.

## Methods

### DNA-NER protein complex

Four 21-mer duplex DNA sequences, encompassing either mutation hotspot or non-hotspot codons in lung tumours were assembled (Table 1). No nuclear magnetic resonance (NMR) structures were available for our adduct bearing sequences; however, an NMR structure for the 10S BPDE DNA adduct in a DNA sequence of the required length was available from the Protein Databank (PDB ID: 1AXO) (Feng et al., 1997). The “Mutagenesis” toolbar in PyMOL and the mutation function on the x3DNA website (http://web.x3dna.org/mutation_file/) were subsequently used to remodel the acquired structure to replicate *KRAS* hotspot and non-hotspot sequences (Schrödinger and DeLano, 2022). All structures were subsequently minimised using the Amber99 force field in the GROMACS module. Modifications in forcefield parameters for to account for 10S bound to guanine were created as detailed in (30,31). This DNA structure was combined with the Rad4 protein, Protein Databank (PDB ID: 2QSG) with our DNA replacing the thymine adducted DNA present in this PDB file (Min and Pavletich, 2007). This was performed using PyMOL’s align function, where this new DNA was aligned with the original DNA and then the original DNA deleted from the file. This combination of guanine adducted DNA and Rad4 protein was then subjected to molecular dynamic simulation, Figure 1. We have termed K12 and K14 sequence with a 10S BPDE adduct in them K12A and K14A respectively and will refer to these throughout the manuscript.

**TABLE 1:** Sequences investigated and their hotspot status. Adducted guanines are highlighted in red. The hotspot status and 10S binding affinity of each site are also depicted.

Codon	Sequence	Hotspot	BDPE affinity
12	GGA|GCT|GGT|GGC|GTA|GGC|AAG	Yes	Strong
14	GGT|GGC|GTA|GGC|AAG|AGT|GCC	No	Strong

### Molecular dynamics simulation

All simulations were carried out with 10 replicates per system, all of which were run using the GROMACS package and Amber99 force field detailed previously (Páll et al., 2020; Menzies et al., 2015; Menzies et al., 2021). Structures were placed in a cubic box, solvated using the explicit water model TIP3P and neutralised with the appropriate number of Na + ions prior to simulation. The Particle mesh Ewald (PME) method was used to treat long-range electrostatic interactions, and a 1.4 nm cut-off was applied to Lennard–Jones interactions. Simulations were carried out using the NPT ensemble, with periodic boundary conditions, at a temperature of 300 K, and a pressure of 1atm. All simulations were performed using three-stage process: steepest descent energy minimisation with a tolerance of 1,000 kJ mol^-1^ nm^-1^, followed by a two-stage equilibration process, each one 50,000 steps in length with a time step integration of 0.002 ps, making a total of 100 ps; and an MD stage run for a total of 100 ns, resulting in a total of 1 μs of sampling per DNA sequence. Simulation files and code used to create them can be found at the following repository: 10.5281/zenodo.16731841.

### Analysis of Rad4-DNA equilibrium trajectories

GROMACS modules were utilised in the evaluation of simulation stability (Hess et al., 2008). Root mean square deviation (RMSD), total energy, pressure and density were considered in this estimation, although radius of gyration (Rg) was also considered given its accurate measure of molecular compactness. Functional parameters, including root mean square fluctuation (RMSF) and solvent accessible surface area (SASA) were also considered in the evaluation of solute dynamics. Data generated between 0 and 10 ns, however, was discarded given that the simulation was still equilibrating. This 10 ns period was selected as it allowed more than enough time for all the simulations to stabilise and therefore enables the parameters of each complex to be measured over a level timeframe. Visualisation of all simulations was carried out in both PyMOL and VMD (Humphrey et al., 1996). Binding free energy was calculated using the GROMACS compatible tool, gmx_MMPBSA (Valdés-Tresanco et al., 2021) and CURVES+ (Lavery et al., 2009) was used to study DNA structural parameters. As with previous analysis techniques employed the 10–100 ns of simulation were analysed and, in this case, we were looking at binding between the protein and DNA (all amino acids within a 3Å radius of DNA were included). Finally, the Residue Interaction Network Generator (RING) was utilised to study protein network interactions, DNA network interactions and bonds between the two. Here 500 frames equally spaced across the 10–100 ns time span were used as input. Statistical testing was carried out in the R environment using appropriate parametric and non-parametric testing.

### Software utilised

For this study we used the following software, with version numbers (Table 2).

**TABLE 2:** Software and versions used in study.

Software name	Version
PyMOL	3.1.4.1
GROMACS	2020
VMD	1.9.4
RING	4.0
CURVES++	2.6
Canal	1.3
gmx_MMPBSA	1.6.4

## Results and discussion

BPDE has long been proposed as the best candidate for the link between smoking and the lung cancer mutational spectrum observed in the *RAS* genes. The precise mechanism underpinning this mutational bias, however, remains elusive. *In silico* methodologies, including MD and free energy calculations, can be used to assess the impact of such distortion on lesion recognition by NER complexes. In order to better understand the relationship between DNA sequence and repair we have studied the interaction between the Rad4 complex and two DNA sequences, one highly repaired in our lung cancer scenario and one often missed leading to a downstream mutational event. We have found differences between the structure of the DNA, the interactions between the protein and DNA in the complex and protein-protein networks. The biological impact of these differences is discussed below.

### Rad4-DNA complexes

Simulations of the Rad4–DNA complex were performed for 10 replicas of 100 ns each, yielding a total sampling time of 1 μs per system. Both control and adducted complexes, i.e., DNA with and without a 10S BPDE adduct at the seventh guanine—were deemed equilibrated after 10 ns, as indicated by stable RMSD values (Supplementary Figure S1).

**FIGURE 2:**
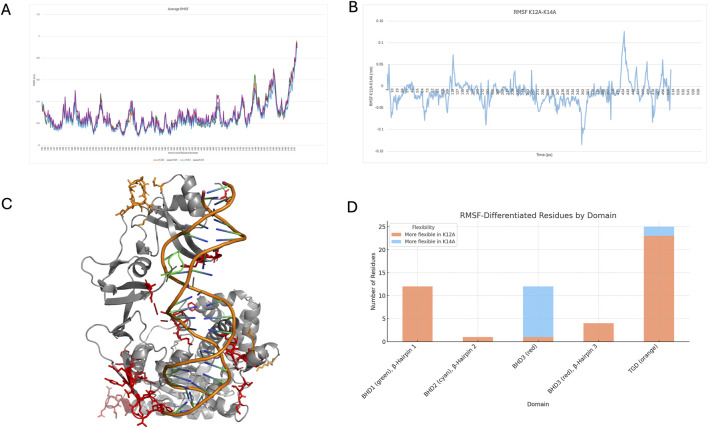
**(A)** average RMSF across each group, **(B)** differences between K12A and K14A average RMSF, **(C)** regions of differences greater than (orange sticks) or less than (red sticks) 0.05 nm, **(D)** number of residues (greater or less than 0.05 nm or more or less flexible) in each of the domains.

Cα RMSF values were calculated over the equilibrated portions and averaged across replicates. Overall, minimal differences were observed across the four groups (Figure 2A); however, small variations between the K12A and K14A systems were identified (Figure 2B). Residues exhibiting RMSF differences ≥0.05 nm or ≤ −0.05 nm were mapped onto the Rad4 structure (Figure 2C). Notably, residues located within the three DNA-binding loops were among those affected, with 23 residues showing a negative difference between residues 130–430, 12 residues between 468–480, one at residue 524, and five between 599–630.

These first 23 residues with this small reduction in flexibility are located within the TGD, which anchors Rad4 to the undamaged DNA strand, potentially emphasising the importance of DNA sequence beyond the damage site. The next group of residues falls within the β-Hairpin Domain 1, specifically the first β-Hairpin, which is the initial loop inserted into the DNA. Residue 524, on the other hand, is on the second β-Hairpin. The remaining residues are situated within BHD3, particularly within β-Hairpin 3. These regions of Rad4 insert into the DNA and are critical for lesion, DNA bending, and eventual base flipping. Consistent with previous findings identifying β-hairpin loops as dynamic elements that facilitate BPDE adduct extrusion, decreased flexibility observed here may have functional significance. All three β-hairpin loops were less flexible in the K14A variant.

A smaller number of residues exhibited a small increase in flexibility in K14A, with two located in the TGD and eleven in BHD3, though none were within the β-hairpin structures. Given the roles of hairpins 2 and 3 in DNA unwinding, the greater or more unstable motion observed in K12A could impair insertion efficiency and hinder proper DNA unwinding. Conversely, increased rigidity in K14A may support more efficient lesion recognition and repair. A full comparison of residues with altered flexibility is shown in Figure 2D. The additional flexibility observed in the TGD and BHD3 regions in K14A may represent a targeted structural adjustment. As the β-hairpins adopt a more rigid configuration to stabilise DNA engagement, neighbouring loops and connecting elements may gain mobility to compensate. This flexibility likely accommodates structural strain from duplex distortion during lesion interrogation and supports the coordinated conformational changes required for efficient base capture and extrusion.

Alongside these we can also see a trend in solvent accessible surface area, groups, in terms of averages and standard deviations can be seen in Supplementary Figure S2, a T-test confirmed no significant difference between groups. However, there is an observable difference between K14 and K14A, than their K12 counterparts, perhaps indicating the protein being more greatly distorted by the adduct and K14 DNA sequence.

### DNA structural differences

To assess DNA structure CURVES + analysis was conducted excluding the first and last DNA base to avoid contributions from highly flexible, loosely held regions that could introduce substantial variance and skew the results. Statistical comparisons between groups were conducted using a T-test. Significant differences (p < 0.05) in several backbone parameters were identified between the K12A and K14A systems. Those which were significant are detailed in Table 3.

**TABLE 3:** Summary of differences between K12A and K14A DNA structural parameter**s** taken as an average from all repeats.

Parameter	p-value	Direction	Interpretation
gammaW	8.44e-18	K14A > K12A	Increased Watson strand torsion
zetaC	1.52e-07	K14A > K12A	Increased Crick strand torsion
epsilC/W	<0.001	K14A > K12A	Enhanced backbone torsional flexibility
alphaC/W	<0.001	Mixed	Entry angle alterations affecting grooves
deltaW	6.79e-04	K14A > K12A	Increased sugar pucker deformation
betaC/W	<0.01	K14A < K12A	Slightly reduced phosphate bending
xdisp	1.07e-02	K14A > K12A	Greater base pair displacement
ampC	3.04e-02	K14A < K12A	Slightly reduced curvature amplitude
majd/W/Curvature/tbend	∼0.03–0.045	Mixed (mostly K14A > K12A)	Increased global DNA curvature

Together with RMSF data, these findings support the conclusion that K12A represents a less flexible model, with reduced disturbance to the DNA structure. This suggests that the BPDE adduct at codon 12 may not sufficiently deform the DNA to trigger Rad4 recognition and repair. Specifically, CURVES + analysis revealed minimal changes in parameters such as twist and backbone torsions in K12A compared to greater distortions observed in the K14A DNA sequences. In K14A, enhanced backbone parameter distortion, affecting groove shape and backbone geometry, may present a more recognisable signal to Rad4, promoting lesion detection and repair initiation. In contrast, the K12A DNA retains features closer to canonical B-DNA, potentially allowing the lesion to evade recognition by appearing structurally normal.

Previously, we demonstrated that 10S BPDE adduction at KRAS codon 12 (K12A) leads to greater distortion relative to other codons and non-adducted controls (Menzies et al., 2021). However, that earlier study examined DNA in isolation, without the repair protein Rad4 present. In contrast, the current simulations incorporate Rad4 binding, and within this context, the K12A DNA region more closely resembles undamaged B-DNA. This difference highlights the critical influence of protein interaction on DNA structure and lesion recognition. While K12A alone induces pronounced distortion, the presence of Rad4 appears to partially stabilise the DNA conformation around the lesion. Importantly, both studies consistently differentiate between highly mutating and less mutating DNA sequences, but including the protein allows for a more comprehensive mechanistic understanding of how DNA structural dynamics and protein engagement collectively determine repair outcomes. These findings emphasise that DNA distortion is highly context dependent; it is not merely the magnitude of distortion, but its nature in the presence of repair factors, that governs lesion recognition and repair efficiency. Within the Rad4 complex, K14A-specific distortions likely provide a stronger signal to trigger repair, whereas the subtler or altered distortion pattern of K12A may impede Rad4 engagement and subsequent lesion processing.

### Protein structure and function

**FIGURE 3:**
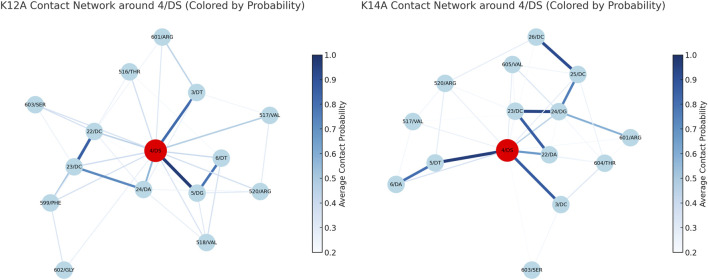
Contact maps from RING data, spanning away from DS4 bases (the adducted base) **(a)**K12A, and **(b)**K14A, thicker and darker lines indicate a stronger connection.

Protein-DNA contact networks were analysed using RING (Del Conte et al., 2024). Comparing K12A and K14A reveals notable differences, particularly around the lesion site (Figure 3). K14A shows contacts with Thr604 and Val605 which are both found in the β-hairpin 3, both K12A and K14A show contacts with Arg601 from this same loop though K12A’s is slightly stronger. K12A, only, has contacts with Phe599. There is a second shift in the β-hairpin 2 where K12A shows stronger or more connections too.

A key observation is the repositioning of β-hairpin 3, marked by changes in Phe599 interactions. In K12A, Phe599 predominantly contacted DNA bases 5′to the lesion, consistent with a compact, damage-engaged configuration. In K14A, Phe599 shifted to engage bases 3′of the lesion, reflecting repositioning of β-hairpin 3 during lesion recognition and repair initiation. This shift parallels a broader reorganisation of Rad4-DNA interactions: in K12A, Rad4 remains tightly anchored through a dense network cantered on the TGD domain, Supplementary Table S1, suggesting persistent lesion engagement. In K14A, the contact network relaxes and shifts, likely facilitating the transition from lesion recognition toward repair progression.

### Energy binding

**FIGURE 4:**
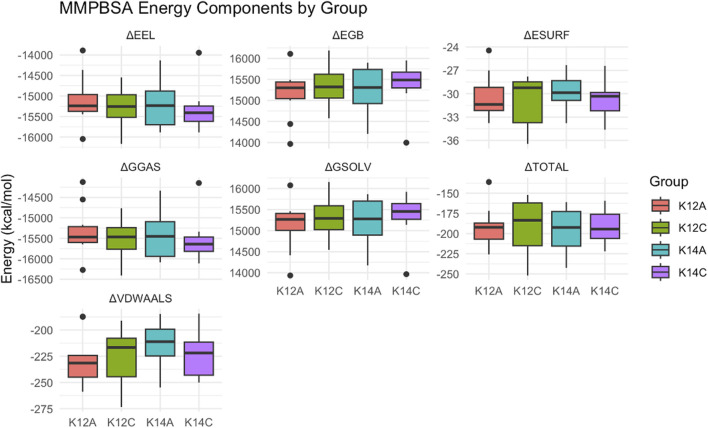
Boxplots for each group and each energy component taken from energy binding data.

No significant differences were observed in total binding energy or its components across the groups; however, K12A exhibited the tightest energy distribution with the least variability (Figure 4). Examination of individual amino acid and DNA base contributions revealed more nuanced differences. Notably, residues within the second and third β-hairpins displayed distinct behaviours. In particular, Phe599, a key lesion-sensing residue, showed persistent, stronger contacts with distorted DNA in the K12A models, consistent with observations from the RING analysis. Previous work by Paul et al. (2020) (Lindahl et al., 2017) demonstrated that β-hairpin 3 plays a critical role in opening damaged DNA, a key early step in lesion recognition. The observed shift in Phe599 contacts in K14A may reflect an enhanced capacity for β-hairpin 3 to engage and destabilise the duplex, aligning with the greater DNA opening seen in K14A compared to K12A.

### Consolidating with the literature

In K14A the 3′ buckling of the 10S-bound guanine nucleobase projects adduct across the transverse axis of the helix, triggering the persistent displacement (flipping) of its partner base. Helical distortion at *KRAS* codon 12, however, was shown to better resist lesion site denaturation, whereby 5′ buckling of its corresponding 10S-bound guanine was shown to project the adduct along the long axis of the duplex. In doing so, native base pairing about the lesion site was weakened, but not fully disrupted in K12A, resulting in transient flipping of the partner base. This transient flipping is less energetically favourable, thereby impeding lesion site denaturation. Discrepancies in lesion orientation were also shown to directly impact base capture. Specifically, in K14A, upon partner base capture, both BHD2 and BHD3 are positioned closer to the duplex compared to K12A. Residues at the domain interface form a deep binding pocket, effectively capturing and anchoring the pre-flipped base through π–π stacking with Phe597. In K12A, however, even at maximal base flipping, the misaligned insertion angle of the BHD3 β-hairpin disrupts pocket formation, leaving it solvent-exposed and devoid of electrochemical attractions, thus diminishing the likelihood of sustained base capture. These findings align with previous studies, which indicate that the efficiency of base capture is influenced by the structural context of the lesion (Lee et al., 2014).

Base flipping is highly uncommon in 10S adducts, with its distortional impact typically limited to disruption of helical rotation, and as such, the shape and size of the grooves. Base capture is, however, highly prominent for the 10R form, given that its covalent incorporation is sustained by intercalation of the helical ladder (Feng et al., 1997). Further simulations could confirm this observation, the consistency of results over an extended period of simulation time (1µs) provides compelling evidence that suggests that base flipping may extend to groove binding adducts under specific topological or sequence contexts. As such, these findings emphasise the way in which adduct conformation may influence the fundamental dynamics of lesion recognition. Notably, impaired base capture at *KRAS* codon 12 may contribute to enhanced resistance to NER, suggesting this as a potential variable influencing repair efficacy.

In all NER systems, the convergence of β-hairpin domains around the lesion forms the mechanical basis for damage recognition and extrusion. The BHD2 β-hairpin, which inserts from the minor groove, plays a critical role in the initial DNA interrogation step. As such, its deletion severely impairs XPC/Rad4 binding, while removal of the BHD3 β-hairpin has only a modest effect (Valdés-Tresanco et al., 2021). Although experimental methods like single-molecule DNA tightrope assays and atomic force microscopy have clarified these functional roles, they are limited in capturing dynamic conformational changes and the impact of pre-existing DNA distortions on protein binding.

MD simulations of protein-DNA complexes overcome these limitations by providing atomistic, time-resolved insights. For example, Paul et al. combined Förster resonance energy transfer with MD to show that the experimentally observed ‘open’ Rad4–DNA complex represents a dynamic ensemble of states (Lavery et al., 2009). Their work revealed alternative DNA-opening pathways and compensatory β-hairpin interactions that maintain robust damage verification, even when one hairpin is impaired. Moreover, free-energy analyses quantified the energetic barriers for base flipping and DNA untwisting, offering mechanistic insights into the slow kinetics of lesion recognition inaccessible to crystallographic or biochemical methods (Lavery et al., 2009). Moreover, MD studies focusing exclusively on DNA have demonstrated that sequence-dependent distortions induced by bulky adducts such as BPDE profoundly affect local DNA structure and flexibility. These alterations, particularly within mutation hotspots of oncogenes like *TP53* and *KRAS*, modify critical helical parameters and disrupt local hydrogen bonding networks, thereby likely modulating minor groove accessibility and the subsequent insertion of β-hairpins, with direct implications for NER efficiency (Hornak et al., 2006; Lindahl et al., 2017). Collectively, these computational investigations provide nuanced mechanistic insights into damage recognition and repair processes that surpass the resolution of conventional experimental methods, underscoring the indispensable role of MD simulations in elucidating the dynamic interplay between DNA sequence context and protein-DNA interactions in NER.

Differences revealed in the initial models incorporating MD-equilibrated Rad4-DNA structures reveal variation in the molecular detail of BHD2 β-hairpin insertion at each codon. Adduct-induced distortions at K14A were shown to differentially stagger traditional Watson-Crick base pairing around the lesion site. Notably, the observed translation of the seventh base step was shown to displace backbone arrangement about 10S aromatics, leading to an increase in minor groove accessibility 5′ to the lesion site. In contrast, at K12A, the 5′ projection of the adduct is shown to inflict contrasting stagger at the sixth base step, leading to constriction of the corresponding minor groove interface. This promoted interaction of the BHD2 β-hairpin with a series of undamaged bases situated distal from the lesion. Using an MD-based approach, Mu et al., report a similar phenomenon whereby inhibited BHD2 insertion via 10S aromatics triggers its translocation one step in the 3′ direction of the lesion-bearing strand (Mu et al., 2018). Such events are likely governed by a lower free energy barrier required to disrupt base pairs at this new entry point, rather than at the original site. These observations highlight the role of free energy in binding pathway progression, supporting the exaggerated repositioning observed in the models.

In GG-NER, stable binding of XPC to damaged duplexes is critical for the recruitment of subsequent repair factors, ultimately ensuring successful repair. Despite this notion, however, the energetic landscapes and associated mechanisms of XPC binding to lesion bound duplexes remains a relatively novel frontier. Recent MD-based studies by Mu et al. from the Broyde group have explored the various binding pathways of the Rad4 to duplex DNA containing the *cis*-B [*a*]P-dG lesion (Mu et al., 2017). The studies reveal that both strong initial binding and a sufficiently low energy barrier for binding pathway progression are crucial factors that regulate the efficacy of lesion recognition by XPC. In the present study we extend this concept by demonstrating that irregular distortion occurring upon 10S adduct formation at *KRAS* codon 12 disrupts traditional binding trajectories. Such events likely increase the energetic threshold to reach the productive binding state, which in turn gives rise to the inherent NER resistance associated with the 10S lesion. This interpretation aligns with the “kinetic gating” mechanism proposed by Chen et al. who suggested that repair-resistant lesions may evade NER detection by presenting an unusually high free energy barrier relative to their residence time, thus decreasing the probability of duplex opening and, consequently, impairing NER efficiency (Min and Pavletich, 2007).

Given the nature of the model used in this present study, the relevance of the observed interactions for describing human-based mutation spectra is of particular significance. Min and Pavletich extensively discuss the sequence and architectural conservation of Rad4 with XPC from multiple species, including humans (Min and Pavletich, 2007). Their findings highlight that, despite demonstrating only a modest sequence similarity of 23%, key residues involved in binding pathway progression, especially those within BHD2 and BHD3, are highly conserved between Rad4 and human XPC. Interestingly, Mu et al. report that critical contacts formed upon helical engagement, particularly those involving residues on BHD3 and the pre-flipped base capture pocket, are also conserved across species (Nem et al., 2015). Despite this however, the 15 amino acid insertion in the BHD2 of human XPC suggests that novel contacts are likely to reside in this region, potentially indicating duplex binding is weaker in yeast XPC models compared to humans given the difference in BHD2 length. Binding assays conducted by the Geacintov group are agreeable with this hypothesis, noting a reduction in the binding of XPC when in solution with the 10S adduct, compared to that of Rad4 (Lee et al., 2014). Nonetheless this, however, the preservation of crucial structural contacts and biochemical specificity validate the use of this model of Rad4 in the absence of a more defined crystal structure for human XPC.

## Conclusion

Our combined structural, dynamic, energetic, and network analyses reveal an explanation the differential repair outcomes between BPDE adducts at *KRAS* codons 12 and 14. Although both lesions feature a bulky adduct, when bound to this Rad4 protein K12A appears to preserve canonical B-form DNA features, showing limited distortion across backbone torsions, sugar puckers, and helical parameters. Further to this, analysis revealed that critical β-hairpin loops, particularly BHD2 and BHD3, exhibit differences in connections to the damaged DNA sequences. Energy decomposition further showed that lesion recognition failure at codon 12 does not relate to global binding differences, but from localised differences at crucial DNA-protein interfaces. Finally, detailed RING analysis uncovered that the poor repair of K12A may stem from suboptimal β-hairpin binding driven by constrained local helical topology.

Together, these results provide evidence that subtle differences in local DNA sequence context drive dramatic differences in NER efficiency. We propose that sequence-encoded structural distortion, rather than lesion chemistry alone, dictates lesion recognisability, binding pathway success, and ultimately mutational hotspot formation within *KRAS* in smoking-related lung cancers. Further work should now be done to expand this with further DNA sequences and adducts, as well as explore the number of BPDE stereoisomers, one of such, 10R forms a different adduct position, intercalating with the DNA rather than sitting in the minor groove. This could impact on Rad4’s ability to bind to the DNA, but we were unable to model it without further Rad4 structure availability.

## Data Availability

The datasets presented in this study can be found in online repositories. The names of the repository/repositories and accession number(s) can be found at https://doi.org/10.5281/zenodo.16731841/.
