# Supplementary material for: Local sequence context at KRAS codons modulates DNA repair efficiency: insights from molecular dynamics simulations
Source: Front Mol Biosci. 2025 Sep 3;12:1654434. doi: 10.3389/fmolb.2025.1654434 (PMC12441607; doi:10.3389/fmolb.2025.1654434)
Supplement: Supplementary file 1 [file DataSheet1.pdf]

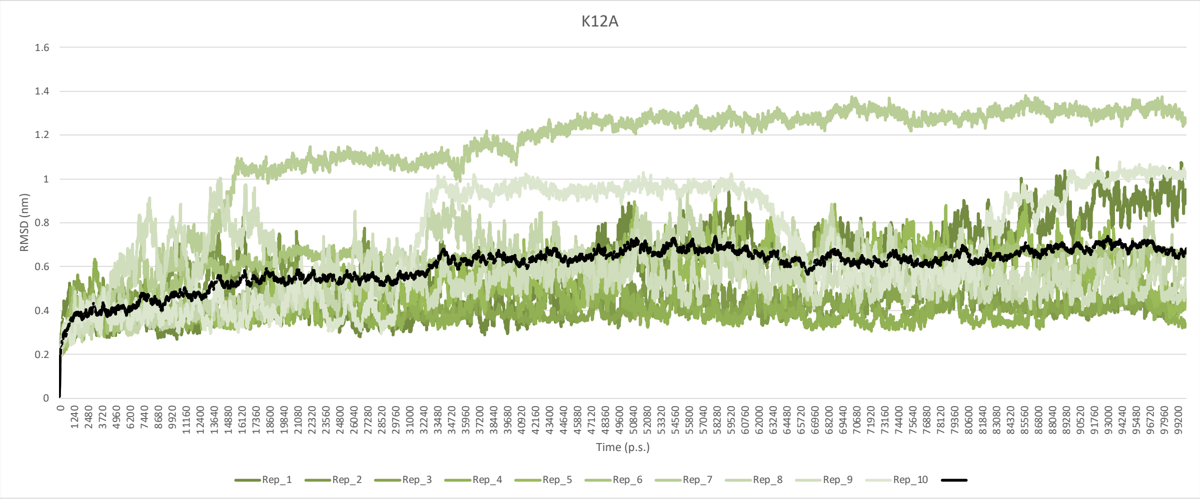
K12A
RMSD (nm)
Time (p.s.)
Rep_1
Rep_2
Rep_3
Rep_4
Rep_5
Rep_6
Rep_7
Rep_8
Rep_9
Rep_10

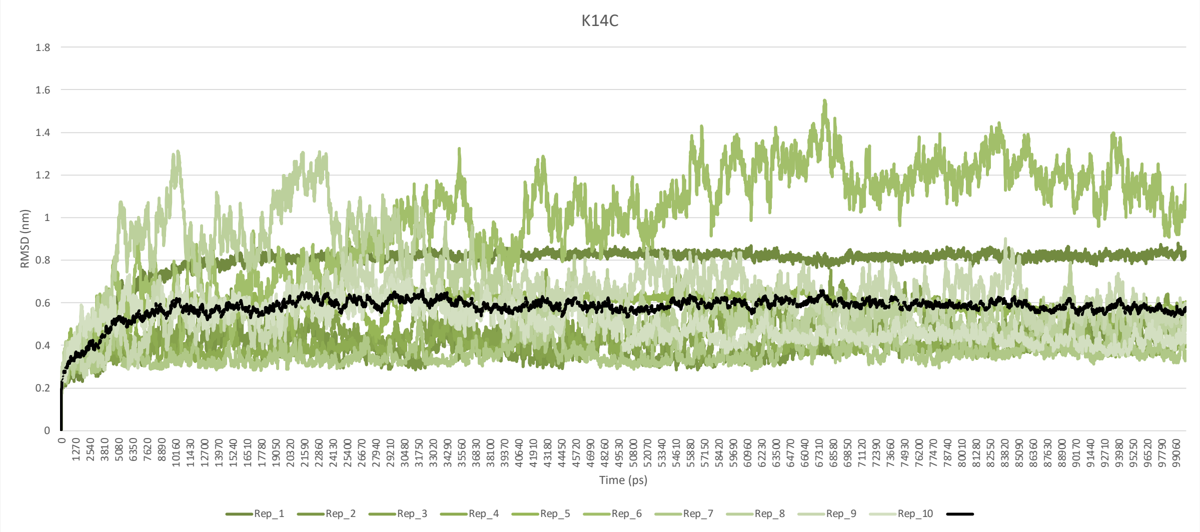
K14C
RMSD (nm)
Time (ps)
Rep_1
Rep_2
Rep_3
Rep_4
Rep_5
Rep_6
Rep_7
Rep_8
Rep_9
Rep_10

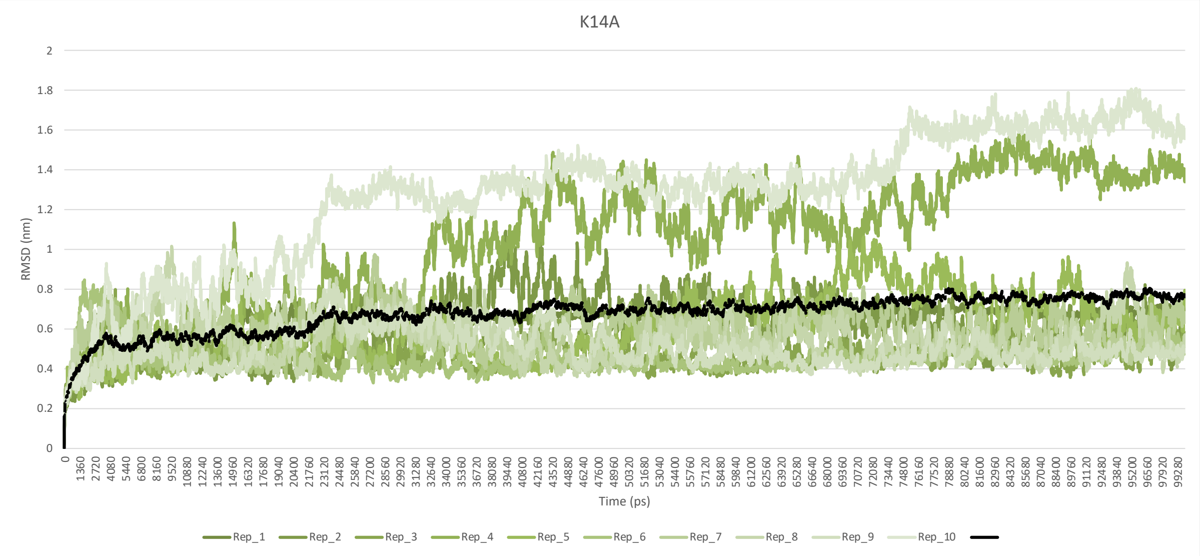
K14A
RMSD (nm)
Time (ps)
Rep_1
Rep_2
Rep_3
Rep_4
Rep_5
Rep_6
Rep_7
Rep_8
Rep_9
Rep_10

Figure S1: RMSD analysis for the equilibrium simulations of RAD-DNA complexes. *Graphs show the time evolution of average RMSD values for each of the 10 100ns simulation replicates. Average values obtained over the course of all replicate simulations is shown in black.*

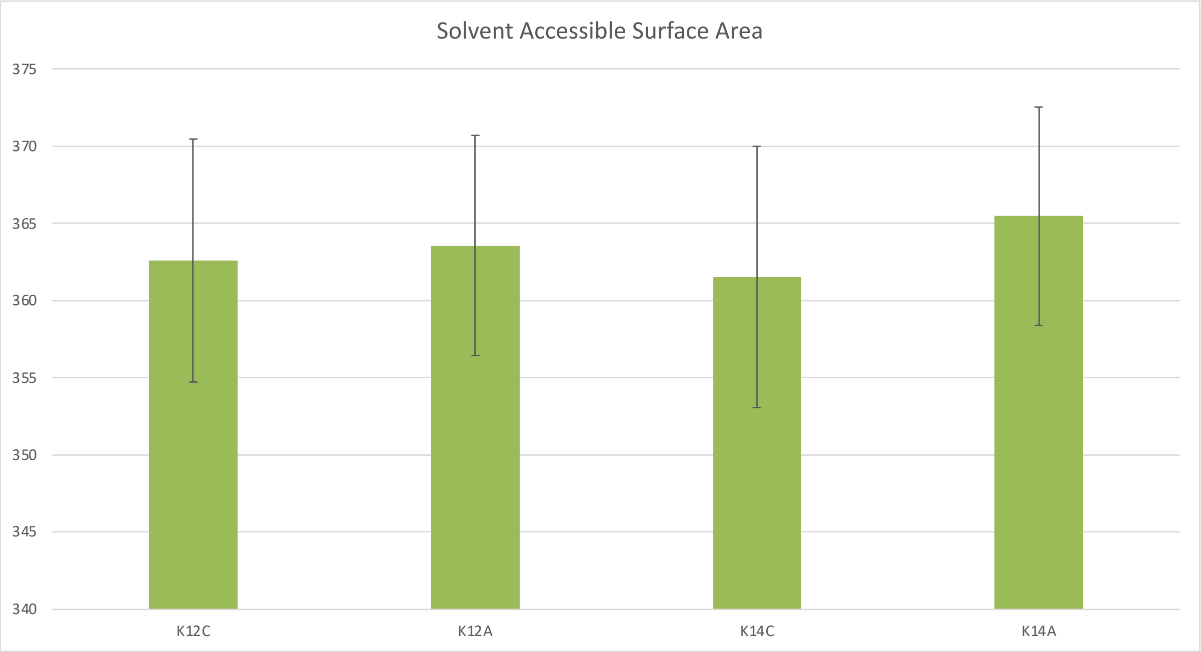

Figure S2: SASA averages per group. *Graph shows the average SASA per group with error bars representing standard deviations.*
